# Biology and Toxicology of Gametes, Embryos, and Cancer Cells in Reproductive Systems

**DOI:** 10.3390/ijms25073639

**Published:** 2024-03-25

**Authors:** Minju Kang, Byeongseok Kim, Youngsok Choi

**Affiliations:** 1 Department of Stem Cell and Regenerative Biotechnology, Konkuk University, Seoul 05029, Republic of Korea; mjjiang@konkuk.ac.kr (M.K.); qufsksrlaqud@naver.com (B.K.); 2 Institute of Advanced Regenerative Sciences, Konkuk University, Seoul 05029, Republic of Korea

Reproduction is the important process of transmitting one’s genetic information to the next generation. The maturation of germ cells and the development process of the embryo after fertilization are greatly influenced by the surrounding environment. This Special Issue aims to provide up-to-date information regarding the biology and toxicity of various cells of the reproductive system (gametes, embryo, and cancer cells). In this article, we introduce background knowledge from papers reported in other journals, the six papers reported in this Special Issue, and the latest trends in the field of reproductive medicine. 

Recently, decreased fertility has emerged as an important social problem due to the aging of women of childbearing age and environmental toxicity, attracting attention in developed countries. Because of this attention, many meaningful studies are being conducted and published in this field.

Germ cells within the reproductive system play an important role in maintaining reproductive function. Normal germ cell development and function are closely related to fertility via the successful development of an embryo. A variety of factors, including environmental toxins, aging, or stress, can cause the DNA damage of germ cells, leading to abnormal metabolism. Wang and colleagues reported that a well-balanced metabolism is essential to the production of high-quality eggs, based on metabolic analysis [1]. The egg becomes activated, meiosis proceeds, and the egg matures. In this process, specific metabolic pathways are regulated, each of which plays a very important role in controlling the epigenetics of the egg before fertilization. 

Pal and Tyler explained that genetic and epigenetic changes such as genomic instability, telomere shortening, the accumulation of DNA damage, and reduced DNA repair capacity increase the frequency of further changes and induce aging [2]. Salonia and colleagues found that somatic cells such as Leydig cells and myoid cells with idiopathic germ cell aplasia in the testis of men deregulated specific genes and pathways and caused the formation of senescent characteristics [3]. This implies that the aging of somatic cells such as Leydig cells affects the normal development of germ cells in the testis. Schumacher et al. reviewed the normal genome maintenance mechanisms in somatic and germ cells, showing that they are completely different in terms of their purposes [4]. They describe that DNA damage in germ cells is more strictly repaired than damage in somatic cells. This is because DNA damage in germ cells can not only cause genetic diseases but also affect the evolution of species, while genetic mutations in somatic cells are only associated with genetic diseases or aging. Somatic cells require maintenance only for the lifespan of an individual, whereas germ cells perpetuate genetic information indefinitely.

Recent studies on genotoxicity under various environmental conditions show that nanoparticles have played an important role in improving our understanding of genetic mutations and cellular damage. Tharmalingam et al. reported the gonadotoxicity of cisplatin in immature human testis, which may be a major factor in explaining chemotherapy-induced infertility [5]. Kohl et al. reviewed the detection of the genotoxicity of various nanomaterials [6]. However, Kim et al. demonstrated the safety of certain nanomaterials by reporting that they do not affect gene expression in the oocytes or embryos, and they are used in various drug delivery systems [7]. 

Hoffmann et al. reported that chromosomal abnormalities show characteristic patterns depending on the age group of the individual. They found that in female group under 20 years of age, aneuploidy increased due to chromosome segregation abnormalities, while in the group over 33 years of age, chromosomal errors occurred more often due to the loss of the chromosome centromere and cohesion between sister chromatids [8]. Marcet-Ortega et al. reported that ATM-CHK2-p53 is used as a surveillance system to detect the persistence of unrepaired DBSs and activate recombination-dependent arrest during the meiotic prophase process in spermatocytes [9].

A variety of research projects are actively being conducted on various factors affecting the reproductive system, in addition to the research mentioned here. This fact illustrates the continued interest in understanding the effects of various environmental factors and aging on the health of reproductive cells. 

We are grateful to the many scientists who took interest in this Special Issue and submitted their research, and we hope that this will be helpful in the development of related fields in the future.
